# Supplementary material for: Genome-Wide Analysis Reveals Novel Regulators of Growth in Drosophila melanogaster
Source: PLoS Genet. 2016 Jan 11;12(1):e1005616. doi: 10.1371/journal.pgen.1005616 (PMC4709145; doi:10.1371/journal.pgen.1005616)
Supplement: S1 Fig — a) Genetic correlation between morphometric traits in males. The two modules of higher correlation observed in females are still visible (bright yellow in the upper left and lower right corners) but the overall clustering is more influenced by the more inaccurately measured smaller veins and areas. b) Cumulative variance explained in male data by increasing number of principal components. As in the female dataset, the first two PCs explain almost 75% of the variance in the data. c) Factor map for the variables. PCs 1 and 2 split the data into two groups. d) Correlation between PCs and traits. PC1 reflects a general size component and PC2 is highly correlated with head/thorax traits, effectively splitting the data in two groups. (PDF) [file pgen.1005616.s001.pdf]

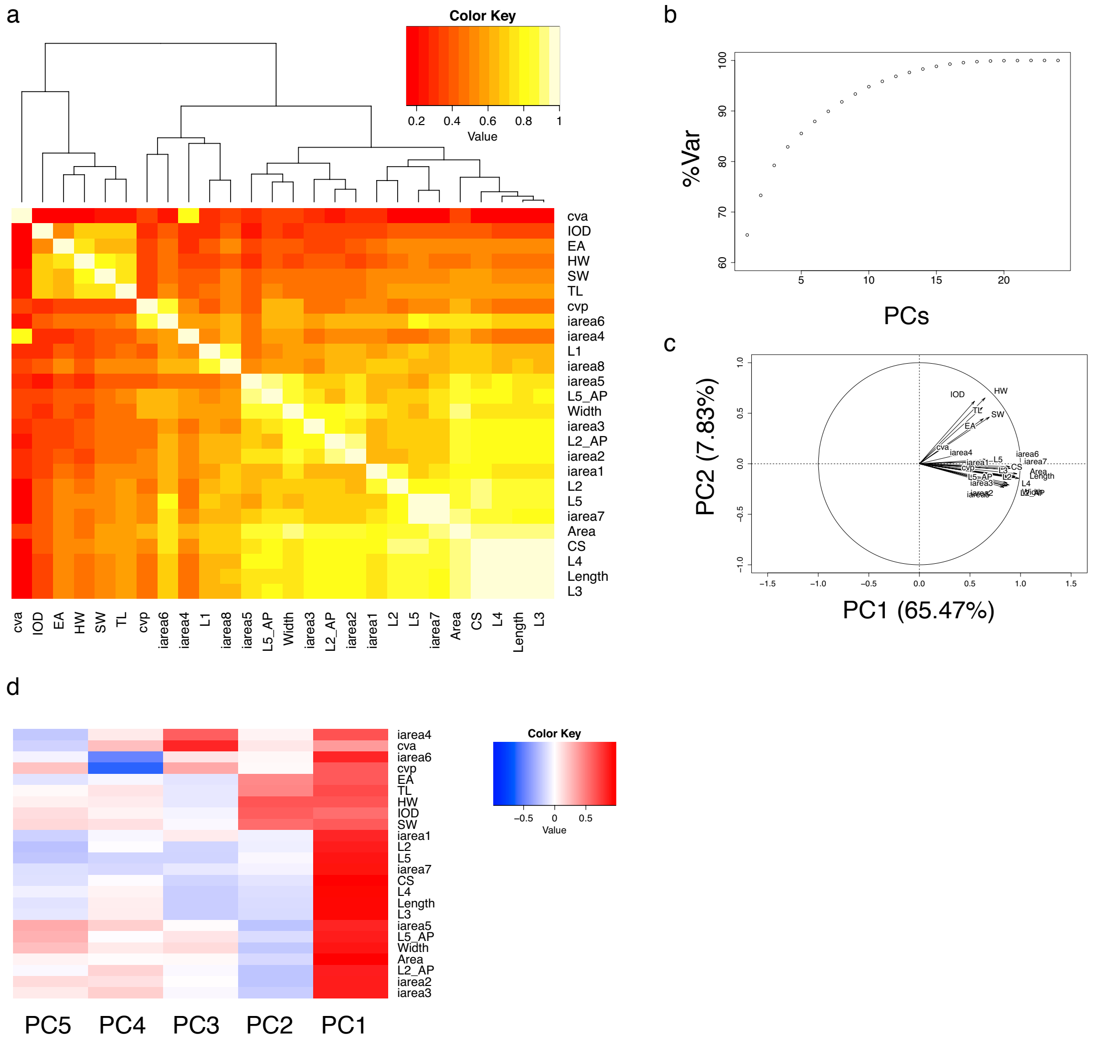

S1 Fig. Analysis of the male dataset. a) Genetic correlation between morphometric traits in males. The two modules of higher correlation observed in females are still visible (bright yellow in the upper left and lower right corners) but the overall clustering is more influenced by the more inaccurately measured smaller veins and areas. b) Cumulative variance explained in male data by increasing number of principal components. As in the female dataset, the first two PCs explain almost 75% of the variance in the data. c) Factor map for the variables. PCs 1 and 2 split the data into two groups. d) Correlation between PCs and traits. PC1 reflects a general size component and PC2 is highly correlated with head/thorax traits, effectively splitting the data in two groups.
