# Supplementary material for: Genome-Wide Analysis Reveals Novel Regulators of Growth in Drosophila melanogaster
Source: PLoS Genet. 2016 Jan 11;12(1):e1005616. doi: 10.1371/journal.pgen.1005616 (PMC4709145; doi:10.1371/journal.pgen.1005616)
Supplement: S2 Fig — Histograms of the estimates for the allometric coefficient b for the relationship between CS and IOD in females (a), in males (b) and between CS and TL in females (c) and males(d). e) Boxplot and individual datapoints of the data in a-d. Red = females and black = males. 95% confidence intervals for b (S1 Table) are very broad for some lines due to few datapoints used for fitting, so these are just very rough estimates for the allometric relationship. Nevertheless there is variation among lines for all evaluated relationships. f) The effect of cosmopolitan inversions on wing size. Lines are plotted according to the number of homozygous inversion arrangements they have: 0 (red) = neither In(2L)t nor In(3R)Mo present, 1 (green) = homozygous for either In(2L)t or In(3R)Mo, 2 (blue) = homozygous for both In(2L)t and In(3R)Mo. Datapoints are individual flies. (PDF) [file pgen.1005616.s002.pdf]

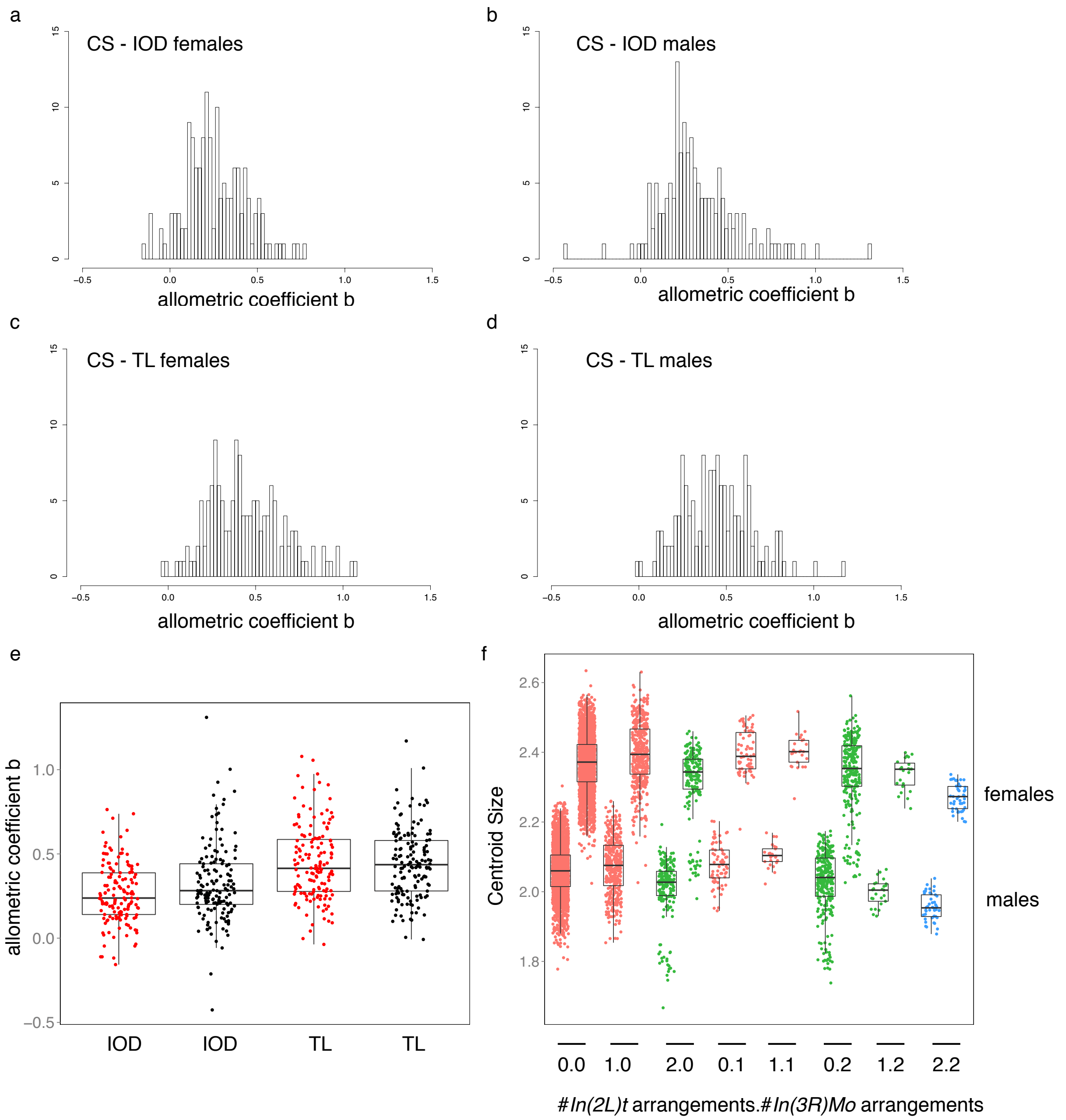

S2 Fig. Allometry and inversions. Histograms of the estimates for the allometric coefficient *b* for the relationship between CS and IOD in females (a), in males (b) and between CS and TL in females (c) and males(d). e) Boxplot and individual datapoints of the data in a-d. Red = females and black = males. 95% confidence intervals for *b* (Supplementary Table 1) are very broad for some lines due to few datapoints used for fitting, so these are just very rough estimates for the allometric relationship. Nevertheless there is variation among lines for all evaluated relationships. f) The effect of cosmopolitan inversions on wing size. Lines are plotted according to the number of homozygous inversion arrangements they have: 0 (red) = neither *In(2L)t* nor *In(3R)Mo* present, 1 (green) = homozygous for either *In(2L)t* or *In(3R)Mo*, 2 (blue) =homozygous for both *In(2L)t* and *In(3R)Mo*. Datapoints are individual flies.
