# Supplementary material for: Genome-Wide Analysis Reveals Novel Regulators of Growth in Drosophila melanogaster
Source: PLoS Genet. 2016 Jan 11;12(1):e1005616. doi: 10.1371/journal.pgen.1005616 (PMC4709145; doi:10.1371/journal.pgen.1005616)
Supplement: S3 Fig — a) The minor allele haplotype of the genome-wide significant cluster is associated with an increased IOD in females. Boxplots of female IOD by genotype at the nine SNPs annotated to kek1. SNPs marked by a star pass Bonferroni correction. Grey = major allele, white = minor allele. b) Lines with the minor haplotype are distributed across all four foodbatches. Black dots = major allele, blue dots = minor allele. The IOD distribution for each foodbatch is plotted for females for the most significant SNP. The distribution is the same for all other SNPs of the cluster as all minor alleles form a haplotype. c) Correlation between p-values from GWAS with normalized IOD (y-axis) and non-normalized iod (x-axis) in females. Axes are on the–log10 scale. d) Several blocks of higher LD are visible in the region 20kb upstream of kek1. Blue = no correlation, orange = complete correlation. (PDF) [file pgen.1005616.s003.pdf]

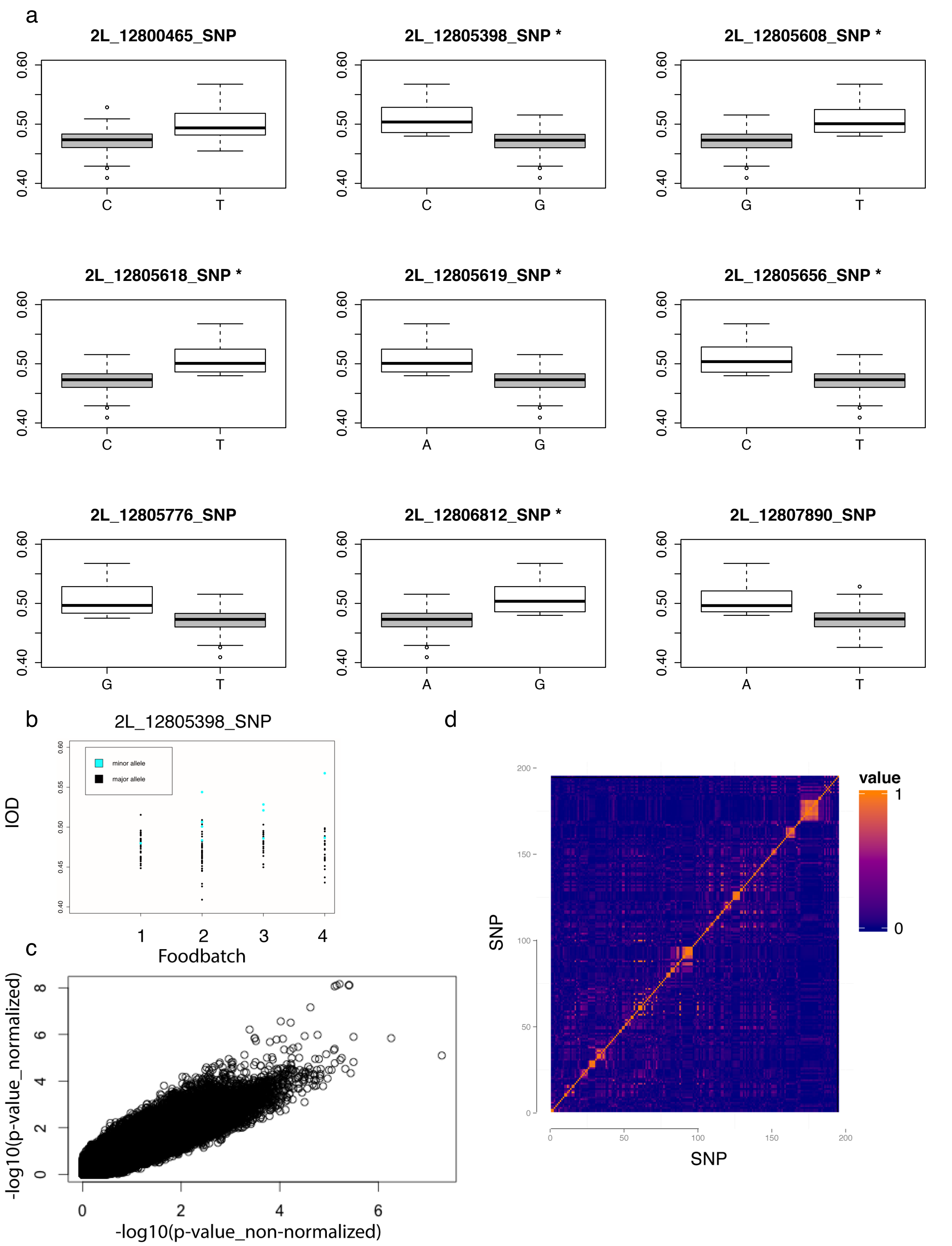

S3 Fig. The minor and major haplotype of genome-wide significant SNPs show differential association with female IOD. a) The minor allele haplotype of the genome-wide significant cluster is associated with an increased IOD in females. Boxplots of female IOD by genotype at the nine SNPs annotated to *kek1*. SNPs marked by a star pass Bonferroni correction. Grey=major allele, white = minor allele. b) Lines with the minor haplotype are distributed across all four foodbatches. Black dots = major allele, blue dots = minor allele. The IOD distribution for each foodbatch is plotted for females for the most

significant SNP. The distribution is the same for all other SNPs of the cluster as all minor alleles form a haplotype. c) Correlation between *p*-values from GWAS with normalized IOD (y-axis) and non-normalized iod (x-axis) in females. Axes are on the –log10 scale. d) Several blocks of higher LD are visible in the region 20kb upstream of *kek1*. Blue = no correlation, orange = complete correlation.
