# Supplementary material for: Genome-Wide Analysis Reveals Novel Regulators of Growth in Drosophila melanogaster
Source: PLoS Genet. 2016 Jan 11;12(1):e1005616. doi: 10.1371/journal.pgen.1005616 (PMC4709145; doi:10.1371/journal.pgen.1005616)
Supplement: S4 Fig — Observed association p-values are–log10 transformed (y-axis) and plotted against the–log10 transformed theoretically expected p-values under the assumption of no association (uniform distribution, x-axis). Centroid size (a), inversion corrected centroid size (b), interocular distance (c), inversion corrected interocular distance (d) and relative centroid size (e). (PDF) [file pgen.1005616.s004.pdf]

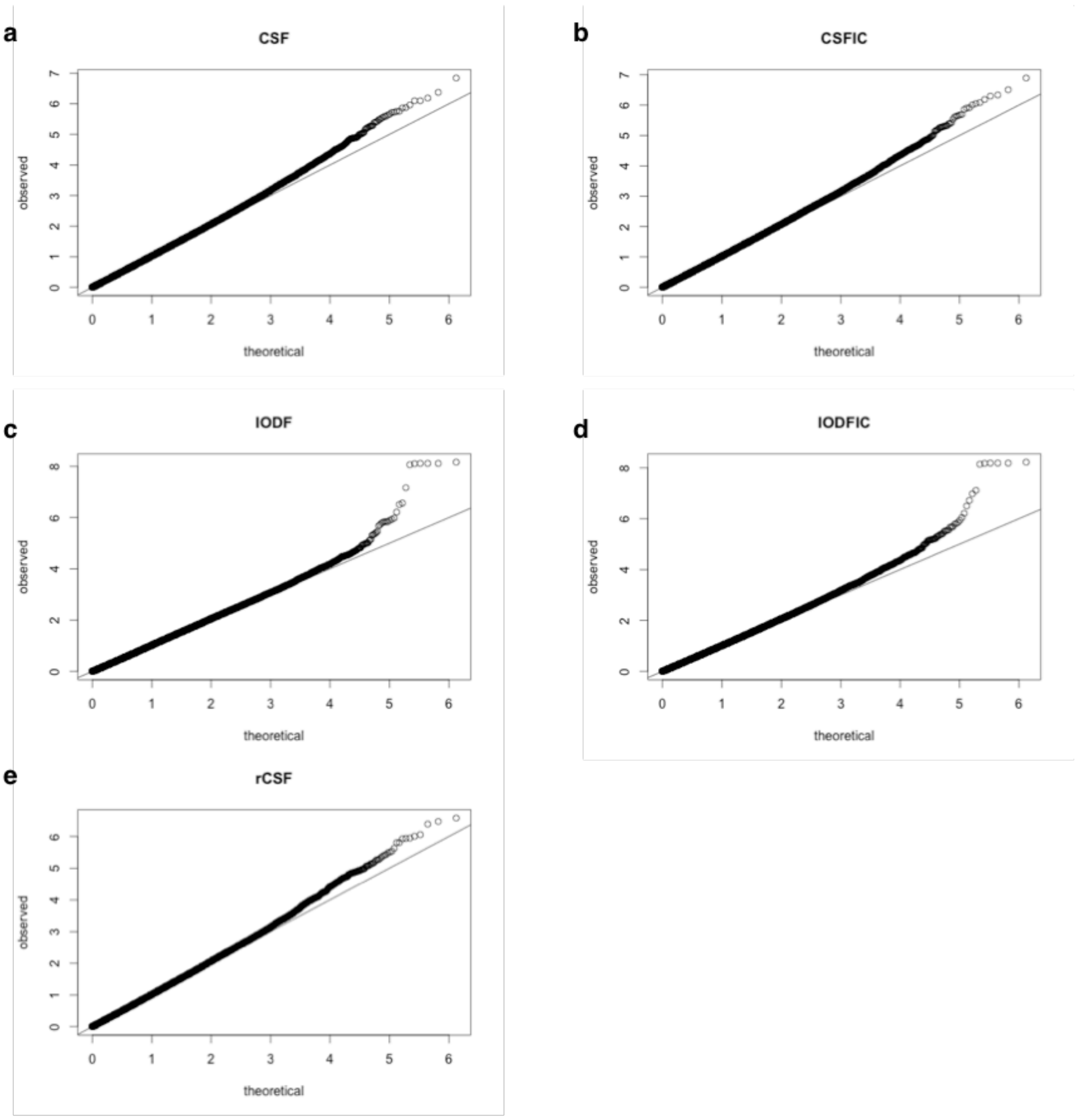

S4 Fig. QQ-plots from GWA in females for all traits show a departure from uniformity of top associations. Observed association *p*-values are –log10 transformed (y-axis) and plotted against the –log10 transformed theoretically expected *p*-values under the assumption of no association (uniform distribution, x-axis). Centroid size (a), inversion corrected centroid size (b), interocular distance (c), inversion corrected interocular distance (d) and relative centroid size (e).
