# Supplementary material for: Genome-Wide Analysis Reveals Novel Regulators of Growth in Drosophila melanogaster
Source: PLoS Genet. 2016 Jan 11;12(1):e1005616. doi: 10.1371/journal.pgen.1005616 (PMC4709145; doi:10.1371/journal.pgen.1005616)
Supplement: S6 Fig — The SNPs are ordered according to chromosome arm (2L, 2R, 3L, 3R, X) and black dividers separate chromosomes. Within one chromosome arm SNPs are ordered according to their position on that chromosome with each tile representing one SNP. The color code is depicted on the right: orange = complete correlation (1) and blue = no correlation (0). Centroid size (a), inversion corrected centroid size (b), interocular distance (c), inversion corrected interocular distance (d) and relative centroid size (e). (PDF) [file pgen.1005616.s006.pdf]

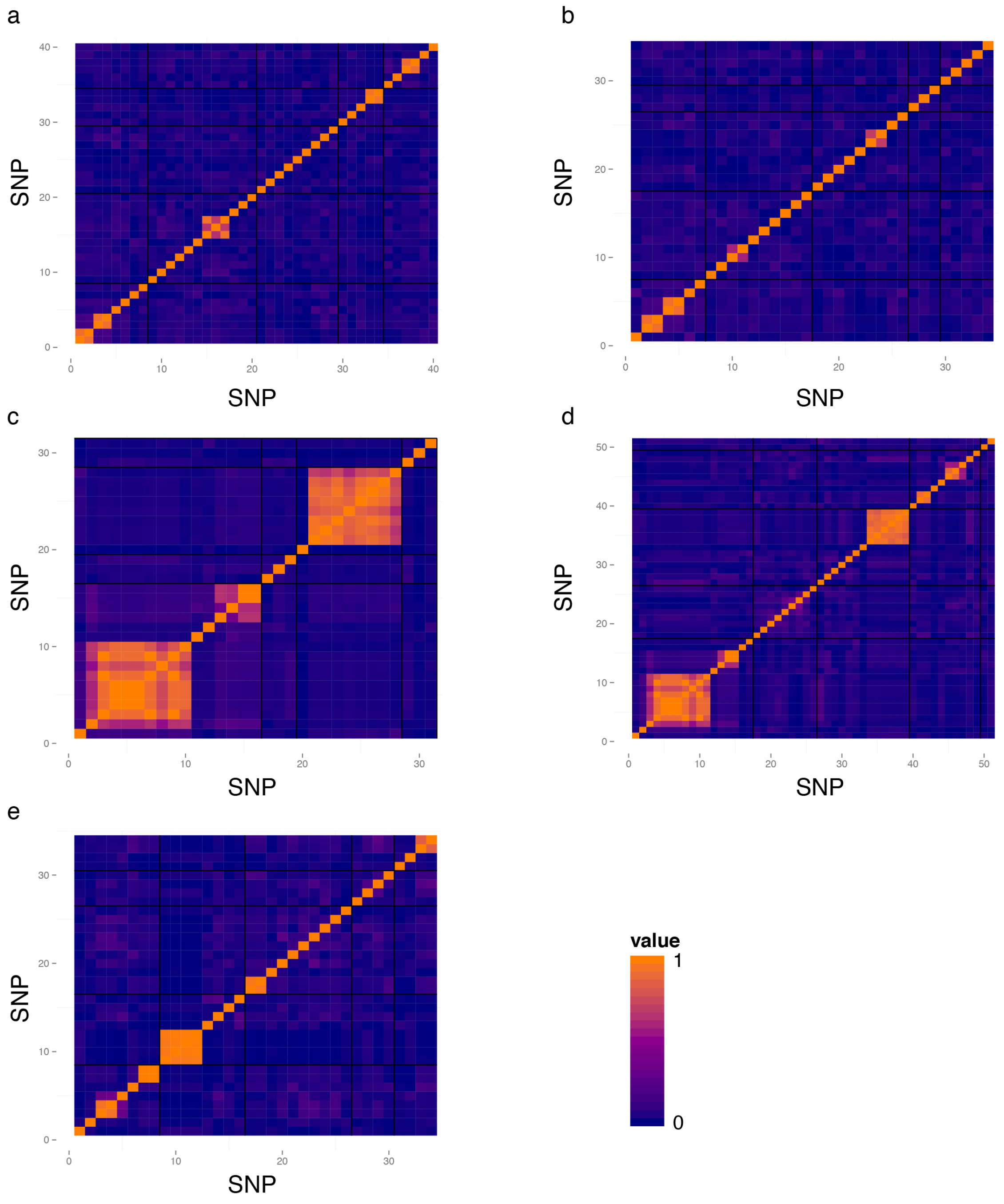

S6 Fig. Correlation between associated (p<10^{-05}) SNPs in females. The SNPs are ordered according to chromosome arm (*2L*, *2R*, *3L*, *3R*, *X*) and black dividers separate chromosomes. Within one chromosome arm SNPs are ordered according to their position on that chromosome with each tile representing one SNP. The color code is depicted on the right: orange = complete correlation (1) and blue = no correlation (0). Centroid size (a), inversion corrected centroid size (b), interocular distance (c), inversion corrected interocular distance (d) and relative centroid size (e).
