# Supplementary material for: Genome-Wide Analysis Reveals Novel Regulators of Growth in Drosophila melanogaster
Source: PLoS Genet. 2016 Jan 11;12(1):e1005616. doi: 10.1371/journal.pgen.1005616 (PMC4709145; doi:10.1371/journal.pgen.1005616)
Supplement: S8 Fig — SNP p-values in females (x-axis) are plotted against their respective p-values in males (y-axis). The Spearman rank correlation is given for each trait and the red lines denote the significance cutoff. a = CS, b = CSIC, c = IOD, d = IODIC, e = rCS. (PDF) [file pgen.1005616.s008.pdf]

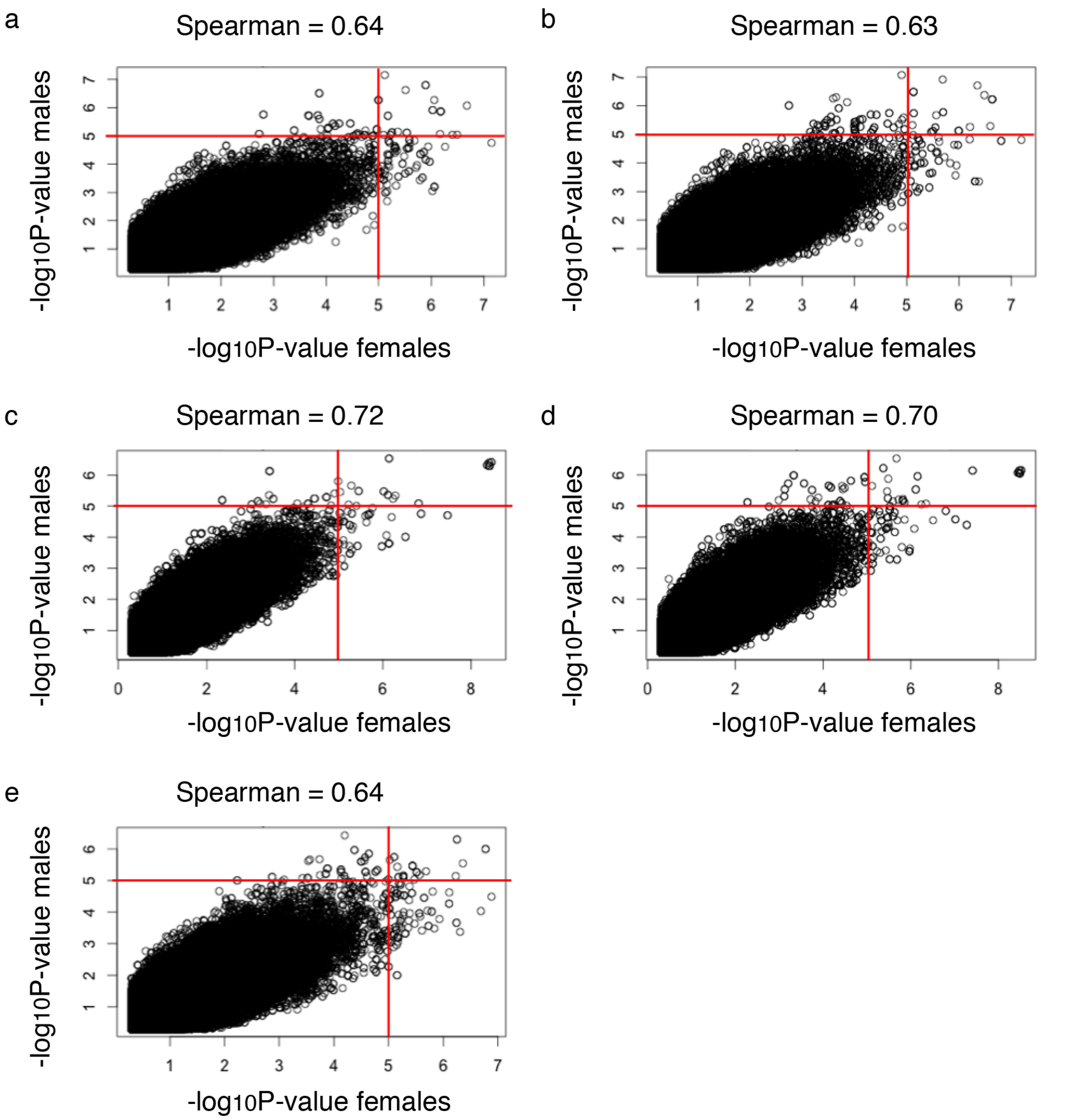

S8 Fig. Correlation of SNP *p*-values between the sexes. SNP *p*-values in females (x-axis) are plotted against their respective p-values in males (y-axis). The Spearman rank correlation is given for each trait and the red lines denote the significance cutoff. a = CS, b = CS_{IC}, c = IOD, d = IOD_{IC}, e = rCS.
