# Supplementary material for: Genome-Wide Analysis Reveals Novel Regulators of Growth in Drosophila melanogaster
Source: PLoS Genet. 2016 Jan 11;12(1):e1005616. doi: 10.1371/journal.pgen.1005616 (PMC4709145; doi:10.1371/journal.pgen.1005616)
Supplement: S9 Fig — Percent change in median wing area compared to CG1315 RNAi upon wing-specific knockdown of the validated candidate genes in males. Only the lines yielding a significant wing size change (p<0.001, Wilcoxon rank sum test) are depicted. Median, 25th and 75th percentile for each are given in S7 Table. (PDF) [file pgen.1005616.s009.pdf]

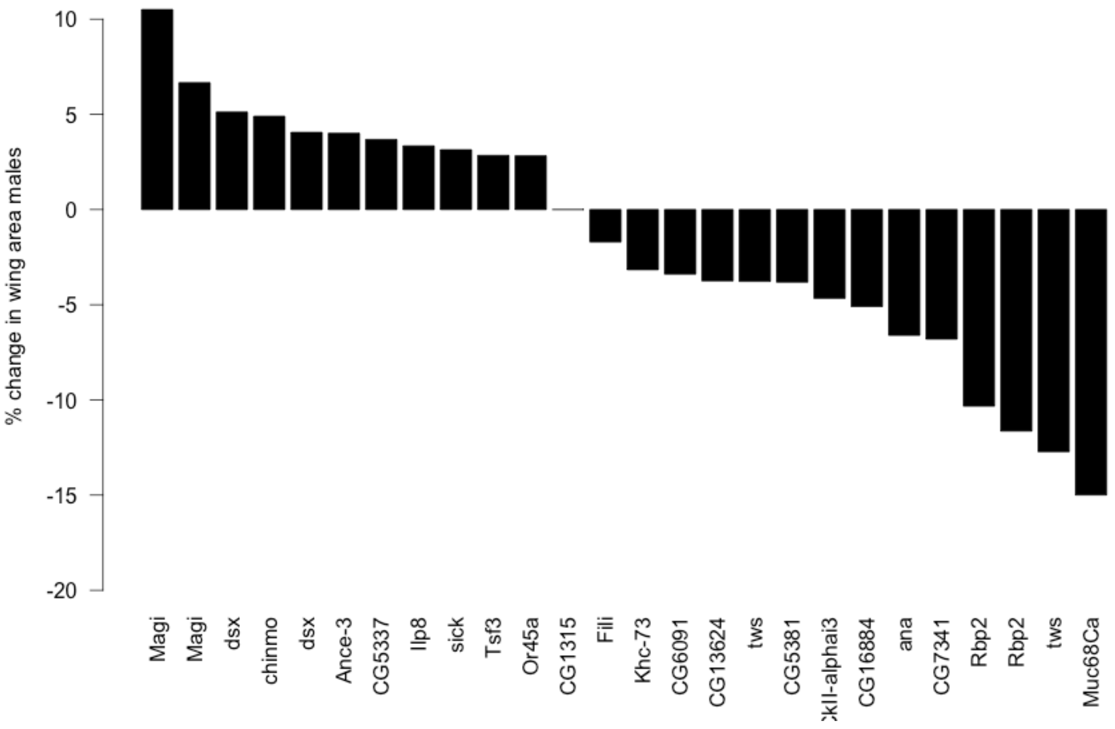

S9 Fig. RNAi knockdown results males. Percent change in median wing area compared to *CG1315* RNAi upon wing-specific knockdown of the validated candidate genes in males. Only the lines yielding a significant wing size change (p<0.001, Wilcoxon rank sum test) are depicted. Median, 25th and 75th percentile for each are given in Supplementary Table 7.
