# Supplementary material for: Genome-Wide Analysis Reveals Novel Regulators of Growth in Drosophila melanogaster
Source: PLoS Genet. 2016 Jan 11;12(1):e1005616. doi: 10.1371/journal.pgen.1005616 (PMC4709145; doi:10.1371/journal.pgen.1005616)
Supplement: S10 Fig — a: -log10 transformed p-value densities of the candidate (black) and combined control (red) data sets. The two p-value distributions differ by a location shift that is not zero (i.e. are not the same); specifically, the–log10 transformed control p-value distribution (red) is shifted towards the left of the–log10 transformed candidate p-value distribution (black) (one sided Wilcoxon rank sum test p = 0.02). b: The distribution of candidate effect sizes (percent change in wing size upon knockdown) is shifted towards positive effect sizes (white boxes), whereas the control knockdown effect size distribution (red) is more centered on 0. The two exceptions at -28% (CG17646) and -42% (CG3704) are lines whose wings not only show a size reduction but also considerable morphological defects (c). N = 43 candidates (white), N = 22 control (red); only data from females was used for these analyses. (PDF) [file pgen.1005616.s010.pdf]

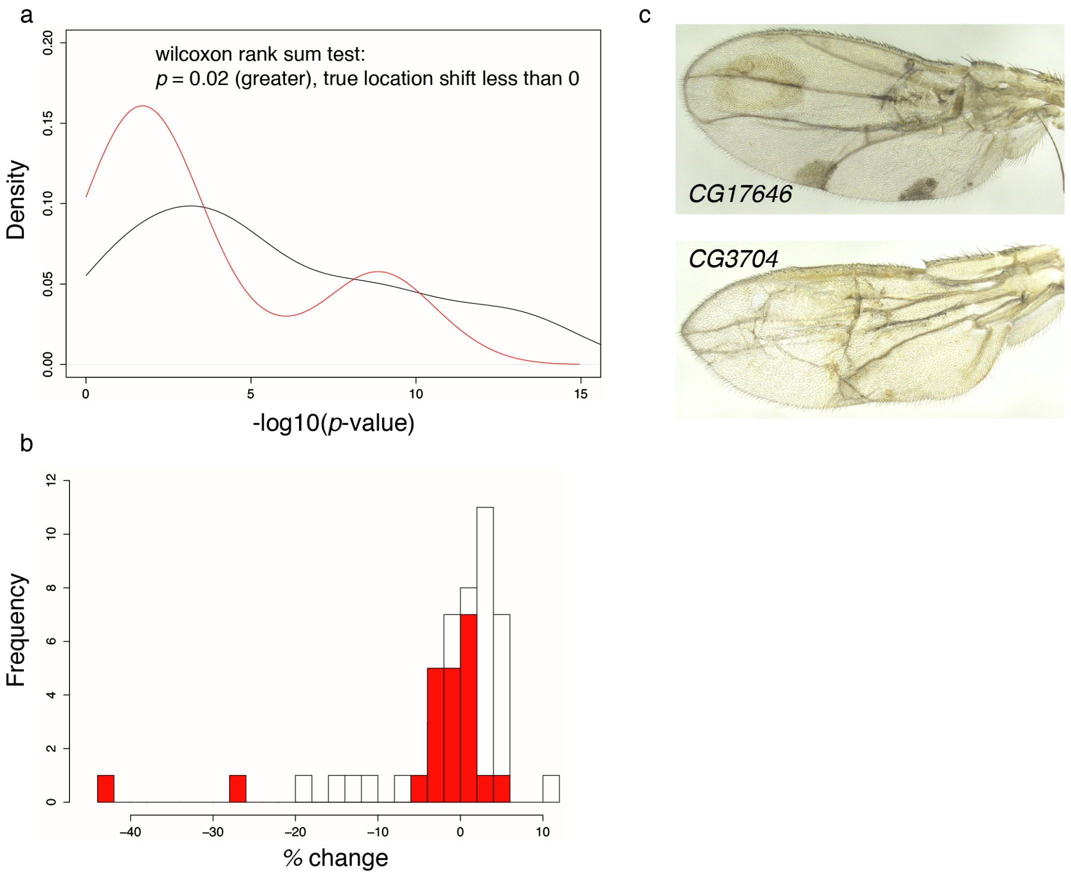

S10 Fig. Comparison of *p*-values and effect sizes between candidate and control RNAi. a: -log10 transformed *p*-value densities of the candidate (black) and combined control (red) data sets. The two *p*-value distributions differ by a location shift that is not zero (i.e. are not the same); specifically, the – log10 transformed control *p*-value distribution (red) is shifted towards the left of the – log10 transformed candidate *p*-value distribution (black) (one sided Wilcoxon rank sum test p = 0.02). b: The distribution of candidate effect sizes (percent change in wing size upon knockdown) is shifted towards positive effect sizes (white boxes), whereas the control knockdown effect size distribution (red) is more centered on 0. The two exceptions at -28% (*CG17646*) and -42% (*CG3704*) are lines whose wings not only show a size reduction but also considerable morphological defects (c). N = 43 candidates (white), N = 22 control (red); only data from females was used for these analyses.
