# Supplementary figures and images for: Genome-Wide Analysis Reveals Novel Regulators of Growth in Drosophila melanogaster
Source: PLoS Genet. 2016 Jan 11;12(1):e1005616. doi: 10.1371/journal.pgen.1005616 (PMC4709145; doi:10.1371/journal.pgen.1005616)

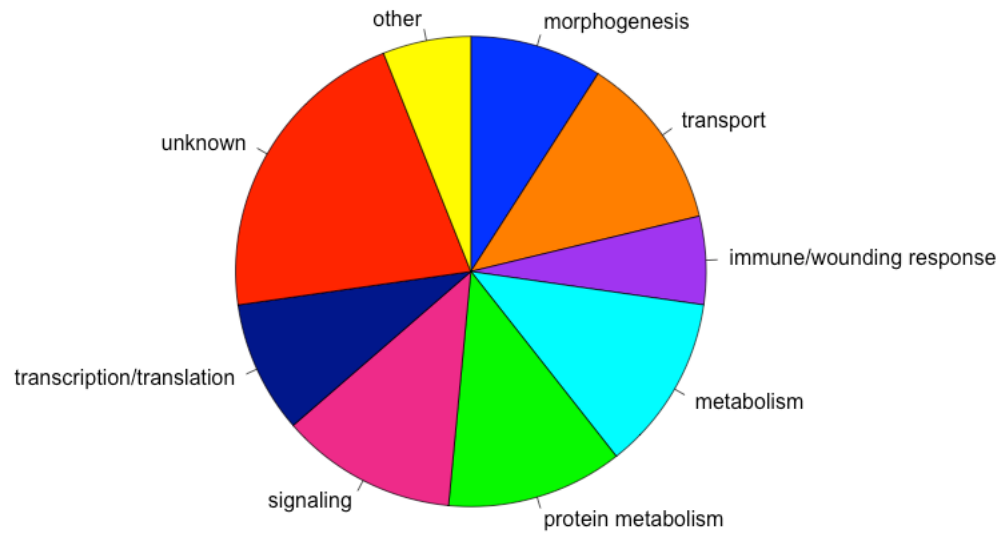

**S11 Fig. Functional annotation of the 33 validated candidate genes based on DAVID GO annotation.**

Supplement: S11 Fig — (PDF) [file pgen.1005616.s011.pdf]
